# LncRNA SNHG6/miR-125b-5p/BMPR1B Axis: A New Therapeutic Target for Triple-Negative Breast Cancer

**DOI:** 10.3389/fonc.2021.678474

**Published:** 2021-05-07

**Authors:** Yufei Lv, Xiaohong Lv, Huike Yang, Xiuying Qi, Xiangchen Wang, Chao Li, Xiaochen Shang, Hongmin Guo, Jianguo Zhang, Yafang Zhang

**Affiliations:** ^1^ Department of Anatomy, Harbin Medical University, Harbin, China; ^2^ Department of Breast Surgery, The Second Affiliated Hospital of Harbin Medical University, Harbin, China

**Keywords:** lncRNA SNHG6, miR-125b-5p, BMPR1B, Triple-negative breast cancer (TNBC), competing endogenous RNAs (ceRNA)

## Abstract

**Background:**

Triple-negative breast cancer (TNBC) is a significant cause of patient morbidity. The exactly pathobiological features of this condition has yet to be completely elucidated.

**Methods:**

Breast cancer data obtained from The Cancer Genome Atlas (TCGA) database were evaluated for lncRNA SNHG6 expression. Normal human breast epithelial cell line (MCF-10A) and other breast cancer cell lines (BT-549, MDA-MB-231, Hs 578t, ZR-75-30, SK-BR-3, MCF-7) were also assessed for lncRNA SNHG6 expressions. Cellular proliferative ability was evaluated with colony formation and CCK-8 assays. The ability of cells to migrate was scrutinized with the wound healing and Boyden chamber cell migration assays. qRT-PCR enabled for detection of lncRNA SNHG6, miR-125b-5p and BMPR1B mRNA expressions. Protein BMPR1B expressions were further assessed using Western Blotting. Direct binding sites between transcripts were determined using dual-luciferase reporter assays. We also constructed a xenograft mouse model to further dissect the vivo implications of lncRNA SNHG6. Ki-67 and c-Caspase-3 expressions were detected using immunohistochemistry staining.

**Results:**

Breast cancer cell lines demonstrated higher lncRNA SNHG6 expressions, particularly TNBC cell lines, in contrast to normal breast epithelial cell lines. This finding coincided with those noted on analysis of TCGA breast cancer data. lncRNA SNHG6 knockdown inhibited TNBC cell proliferation, migration, while promoted cell apoptosis. Furthermore, suppressed lncRNA SNHG6 expressions resulted in lower tumor weights and volumes in a xenograft mouse model, as evidenced by Ki-67 and c-Caspase-3 expression profiles in tumor tissues. miR-125b-5p and lncRNA SNHG6/BMPR1B both possessed direct binding sites for each other which was validated utilizing a dual-luciferase reporter assay. Decreasing lncRNA SNHG6 expression in TNBC cells upregulated miR-125b-5p expression. Another side, inhibiting miR-125b-5p upregulated BMPR1B expression in these cells. Moreover, knocking down lncRNA SNHG6 downregulated BMPR1B expression in TNBC cells, and the finding was rescued in cells which were exposed to miR-125b-5p inhibitor. Downregulating miR-125b-5p mitigated the effect of suppressing lncRNA SNHG6 on TNBC cell proliferation, migration, and apoptosis.

**Conclusion:**

Downregulation of lncRNA SNHG6 could inhibit TNBC cell proliferative, migratory capabilities and promote apoptosis capability, likely through modulation of the miR-125b-5p/BMPR1B axis. This axis may be targeted in formulating new therapies for TNBC.

## Introduction

The second most frequently encountered reason of cancer-associated death in women around the world is due to breast cancer (1). Triple negative breast cancer (TNBC) is the most malignant subtype which makes up roughly 10%–20% of all diagnosis of cancer in this organ (2). Its incidence rate is nearly equal to its mortality rate, with this debilitating disease diagnosed in an increasingly younger population over the last few years. The lack of typical receptors such as human epidermal growth factor receptor 2 (HER2), progesterone receptor (PR) and estrogen receptor (ER) on this cancer subtype precludes usage of currently available targeted therapeutic agents (3). Therefore, further research on uncovering other potential treatment targets in TNBC is much needed.

Long non-coding RNAs (lncRNAs) are transcripts which possess more than 200 nt and have been found to interact in a myriad of biological processes and diseases, including cancer (4–6). For example, 1484 differentially expressed lncRNAs were discovered in lung cancer, of which 535 were upregulated and 949 were downregulated (7). 172 lncRNAs in endometrial carcinoma were determined to be differentially expressed in contrast to normal endometrial samples (8). Microarray analysis revealed a total of 2925 dysregulated lncRNAs in TNBC samples (9). However, the exact effects of dysregulated lncRNA expressions have yet to be fully determined. Both breast cancer cell lines and samples, especially those of TNBC, have been found to harbor high expressions of lncRNA H19, which has been implicated to increased rates of metastasis and tumorigenesis (10). Moreover, lncRNA H19 may promote breast cancer tamoxifen resistance through modulation of the SAHH/DNMT3B axis (11). The LncRNA HOX transcript antisense RNA (HOTAIR) is upregulated in breast cancer cell lines and samples, which has been associated to the progression of breast cancer due to its action on the miR-20a-5p/HMGA2 axis (12). One lncRNA of interest is the oncogenic small nucleolar RNA host gene 6 (SNHG6), which is aberrantly expressed in cancers such as glioma, hepatocellular carcinoma as well as in lung and colorectal cancers (13–16). Raised SNHG6 expressions was intricately related to poorer overall survival in cancer patients (17). There is an upregulation of lncRNA SNHG6 in high-grade and progesterone receptor-positive breast cancer tissues, which may be associated to breast cancer cell migration and epithelial-mesenchymal transition (EMT) (18). Nevertheless, the role of lncRNA SNHG6 in TNBC has yet to be discovered.

The current study uses qRT-PCR to assess quantities of lncRNA SNHG6 in breast cancer cells. SNHG6 was overexpressed in both MDA-MB-231 and BT-549 two kinds of cell lines. The CCK-8, wound healing, colony formation, cell migration and TUNEL assays along with the xenograft mouse model allowed for our study group to scrutinize the consequences of SNHG6 on TNBC cellular proliferation, migration and apoptosis. Gene transcripts were also assessed *via* qRT-PCR. Western Blot was utilized in detection of functional protein bone morphogenetic protein receptor type 1B (BMPR1B) expression. We also used dual-luciferase reporter assay and functional rescue assays to verify the crucial role of SNHG6 in TNBC.

## Materials and Methods

### Cell Culture and Transfection

The Shanghai Zhong Qiao Xin Zhou Biotechnology Co., Ltd (Shanghai, China) supplied MDA-MB-231 cells. The Cell Bank of the Chinese Academy of Sciences (Shanghai, China) supplied human TNBC cell line (BT-549) and normal breast epithelial cell line (MCF-10A). MDA-MB-231 cells were maintained using DMEM (Gibco, Invitrogen, CA, USA) while BT-549 cells were maintained using RPMI 1640 (Gibco, Invitrogen, CA, USA). Both cell cultures contained 100 U/mL streptomycin/penicillin and 10% fetal bovine serum (Beyotime, Shanghai, China). MEGM SingleQuots (Lonza, Walkersville, MD, USA) was used to culture MCF-10A cells. All the aforementioned cell lines were kept in a T25 tissue culture flask and incubated under 5% CO _2_ at 37°C.

The siRNA sequence targeting lncRNA SNHG6 was synthesized by Ribobio (Guangzhou, China). Negative controls were designated as si-NC. miR-125b-5p expressions were inhibited by an inhibitor produced by the same company. All transfections were carried out in strict compliance to manufacturer protocols. The siRNA targeting lncRNA SNHG6 was as follows: GCGGCATGTATTGAGCATA.

### Cell Counting Kit-8 (CCK-8) Assay

After a 24-hour transfection period, all cells were plated onto 96-well plates at a concentration of 4 × 10^3^ cells per well. Cellular activity was detected at 24 h, 48h, 72h, 96h. This was done by adding a tenth of a volume of Cell Counting Kit-8 reagent (bimake, Houston, USA) into each well before the entire plate was incubated for 1 to 1.5 hours in an incubator until cell media turned color. Each group possessed a negative control well. The OD value was detected at 450 nm using an enzyme-labeled standard instrument. Proliferation curves were constructed using the average OD values. All assays were performed in triplicate.

### Colony Formation Assay

TNBC cells (1000 cells per well) which were transfected for 24 hours were transferred onto a 6-well culture plate and underwent a 1 week incubation period with the culture medium replaced every three days. Colonies were then fixed using 4% histiocyte fixative (Solarbio Sciences, Beijing, China) and stained with 0.1% crystal violet methanol solution (Solarbio Sciences, Beijing, China). All colonies were counted and photographed.

### Wound Healing Assay

TNBC cells which underwent a 24-hour transfection period were placed in a 6-well plate at a concentration of 7 × 10^5^ cells per well and cultured in an incubator for one day. Pipette tips were used to create three separate wounds in each well of the 6-well plates and the wound was photographed immediately (at 0 h). The cells were rinsed and cultured for another 48 h before being photographed again. The ImageJ software was used to measure the degree of wound closure.

### Transwell Assay

The upper chamber of a transwell assay was used to house transfected cells (1 × 10^5^) in 200 μl FBS-free media. Lower chambers contained 600 μl medium mixed with 30% FBS. The system was left alone for one day. After this, cells which were found to have migrated into the bottom chamber were fixed with 4% histiocyte fixative (Solarbio Life Sciences, Beijing, China) and stained with 0.1% crystal violet methanol solution (Solarbio Life Sciences, Beijing, China).

### TUNEL Assay

To analyze cell apoptosis, terminal deoxynucleotidyl transferase mediated dUTP nick-end labeling (TUNEL) assays were performed with Tunel Apoptosis Detection Kit (Fluorescence - Green Light) (Wanleibio, Shenyang, China) according to the manufacturer’s instructions. FITC-labeled apoptotic cells were observed under the fluorescence microscope (OLYMPUS BX51, Japan).

### Quantitative Real-Time Polymerase Chain Reaction (qRT-PCR)

The TRIzol reagent (Ambion, 15596026, CA, USA) allowed for total RNA extraction from TNBC cells according to the manufacturer instructions. cDNA was reverse transcribed using the PrimeScript RT reagent Kit (Perfect Real Time, Japan) (Takara, RR037A). qRT-PCR assay was operated using the TB Green Premix Ex Taq II (Tli RNaseH Plus) (Takara, RR820A, Japan). Each experiment was repeated in triplicate. The following qRT-PCR assay primer sequences were used: lncRNA SNHG6: CGGCATGTATTGAGCATATAGGT (forward) and CACACTTGAGGTAACGAAGCAGA (reverse); BMPR1B: CCAAAGGTCTTGCGTTGTAAA (forward) and ACCCAGAGTCATCC TCTTCTATCA (reverse). miRNA qRT-PCR Primer Sets designed by RiboBio (Guangzhou, China).

### Western Blot Assay

Both MDA-MB-231 and BT-549 cells were treated with RIPA lysis buffer (Beyotime, Shanghai, China) supplemented with 1%PMSF for total protein extraction. A 10% SDS-PAGE gel was used to separate component proteins before they were immunoblotted onto a PVDF membrane (Bio-Rad, CA, USA). 5% skim milk powder was used to block endogenous reactions and the entire membrane was incubated with primary antibodies against BMPR1B (1:1000) (Affinity Biosciences, OH, USA), Bax (1:800), Bcl-2 (1:800), c-Caspase-3 cleaved (1:800) and β-actin (1:2500) (Bioss, Beijing, China) at 4°C overnight and rewarmed for an hour in the next morning. Membranes were rinsed thrice with TBST before being re-incubated with secondary antibodies (1:5000) (Bioss, Beijing, China) for 1.5h at 25°C. Three final rinses were then performed prior to examination using an Automatic Chemiluminescence Imaging Analysis System (Tanon, Shanghai, China).

### Animal Experiments

The Harbin Medical University Animal Care and Use Committee reviewed approved all animal experimental protocols to ensure they were in accordance to protocols stipulated by the National Institutes of Health. Nude mice were purchased from Beijing Weitonglihua Laboratory Animal Technology Co., Ltd. (Beijing, China) and inoculated with MDA-MB-231 cells transfected with either si-SNHG6 or si-NC. Tumor volumes were assessed weekly. All mice were sacrificed at 28 days post-inoculation and tumors were dissected for further assessment.

### Immunohistochemical Staining (IHC)

The IHC staining of paraffin-embedded tissues was operated following the right steps. Primary antibodies against Ki-67 and c-Caspase-3 were covered over tissues at 4°C for overnight. The next morning, secondary antibodies were covered over tissues at room temperature for 50 minutes. After adding DAB chromogenic solution, the tissues were examined under microscope.

### The Prediction of RNA Binding Sites and Dual-Luciferase Reporter Assay

Sequences containing the miR-125b-5p binding site on the 3’-UTR of lncRNA SNHG6 or the 3’UTR of BMPR1B were amplified by the ABI Gene Amp PCR System 2400. The amplified products were merged into the psiCHECK™-2 Vector to construct the lncRNA SNHG6 and BMPR1B wild-type (WT) and mutant-type (MUT) reporter plasmids, respectively. A dual-luciferase reporter assay system (Promega, Fitchburg, USA) was utilized for detection of luciferase activity.

### Data Analysis

All data was determined in terms of mean ± SEM. The ANOVA or Student’s t-test was used to determine differences between multiple or two groups, respectively.

## Results

### The Expression of lncRNA SNHG6 in Breast Cancer

**Figure 1:**
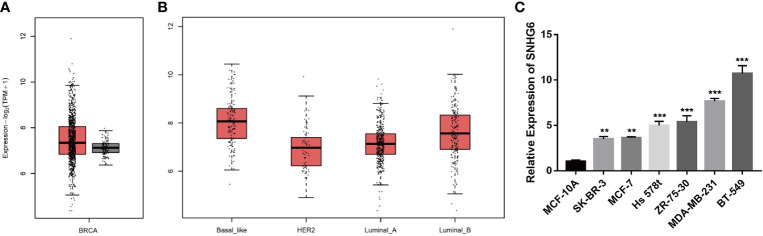
lncRNA SNHG6 expression across breast cancer databases and cell lines **(A)** Levels of SNHG6 expression were detected in breast cancer and normal samples in TCGA data base. **(B)** Levels of SNHG6 expression were detected in different breast cancer subtypes. **(C)** lncRNA SNHG6 expression was raised in breast cancer cell lines in contrast to normal breast epithelial cell line. **P < 0.01, ***P < 0.001 vs. MCF-10A. n = 3.

We initially evaluated lncRNA SNHG6 transcription levels in breast cancer studies based on data from TCGA with the Gene Expression Profiling Interactive Analysis 2 (GEPIA 2) online tool (http://gepia2.cancer-pku.cn/). We found that SNHG6 expressions appeared to be raised in samples of breast cancer in contrast to healthy breast tissue samples (**Figure 1A**). Additionally, “Basal-like” samples were noted also have high expressions of SNHG6 compared to those of other types (**Figure 1B**). lncRNA SNHG6 expressions were then characterized in MCF-10A, BT-549, MDA-MB-231, Hs 578t, ZR-75-30, SK-BR-3 and MCF-7 cells. All breast cancer cell lines were significant for high lncRNA SNHG6 expressions in comparison to normal mammary epithelial cell line. lncRNA SNHG6 expression was also found to be elevated in TNBC cell lines (BT-549, MDA-MB-231, Hs 578t) in contrast to non-TNBC cell lines (ZR-75-30, SK-BR-3, MCF-7) (**Figure 1C**). TNBC cell lines (MDA-MB-231 and BT-549) with higher lncRNA SNHG6 expression were selected for additional experiments.

### lncRNA SNHG6 Inhibition Slowed Proliferation of TNBC Cells

**Figure 2:**
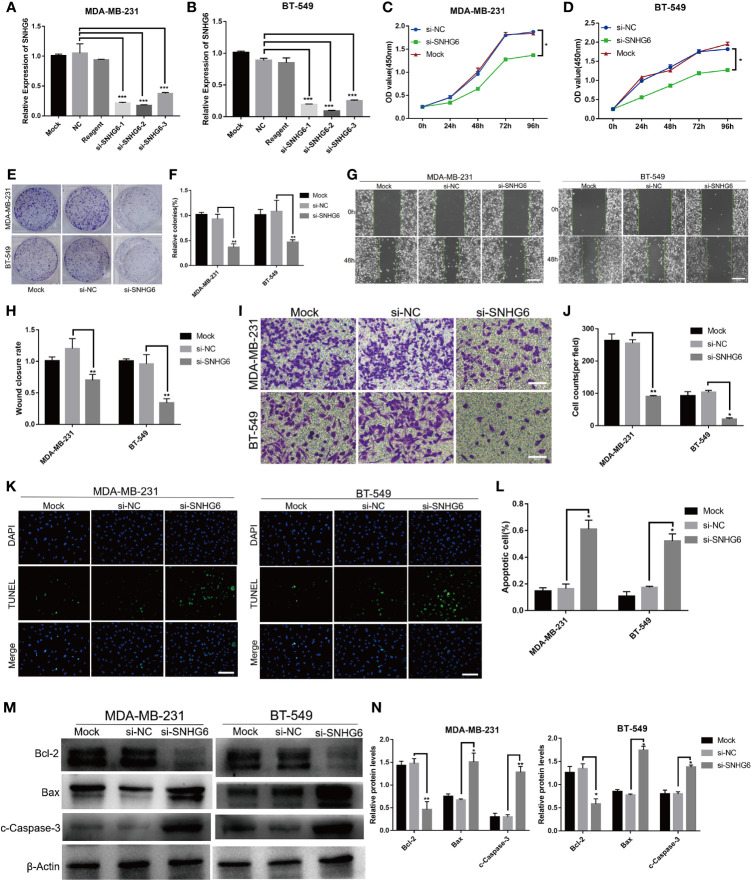
Suppression of lncRNA SNHG6 attenuated TNBC cell migration and proliferation, while promoted cell apoptosis. **(A)** The siRNA targeting lncRNA SNHG6 inhibited lncRNA SNHG6 expression in MDA-MB-231 cells. **(B)** The siRNA targeting lncRNA SNHG6 inhibited lncRNA SNHG6 expression in BT-549 cells. **(C)** lncRNA SNHG6 inhibition suppressed the proliferation of MDA-MB-231 cells. **(D)** lncRNA SNHG6 inhibition suppressed the proliferation of BT-549 cells. **(E)** Representative colony formation images of MDA-MB-231 and BT-549 cells. **(F)** lncRNA SNHG6 inhibition reduced colony-forming of both two kinds of cells. **(G)** Representative wound healing assay images of MDA-MB-231 and BT-549 cells. Magnification×40; Scale bar, 200 μm. **(H)** The inhibition of lncRNA SNHG6 suppressed migration capability of MDA-MB-231 and BT-549 cells. **(I)** Boyden chamber cell migration assay in MDA-MB-231 and BT-549 cells, respectively. Magnification ×100; Scale bar, 100 μm. **(J)** lncRNA SNHG6 inhibition diminished migration capability of both two kinds of cells. **(K)** TUNEL assay in MDA-MB-231 and BT-549 cells, respectively. Magnification×100; Scale bar, 100 μm. **(L)** lncRNA SNHG6 inhibition promoted apoptosis capability of both two kinds of cells. **(M)** The protein expression of Bcl-2, Bax and c-Caspase-3 in MDA-MB-231 and BT-549 cells. **(N)** lncRNA SNHG6 inhibition induced the protein expression level of Bcl-2 decreased while the level of Bax and c-Caspase-3 increased. *P < 0.05, **P < 0.01, *** *P* < 0.001 vs. si-NC; n = 3.

SiRNA targeting lncRNA SNHG6 was used to knock down lncRNA SNHG6 expression *in vitro*. Three different sequences (si-SNHG6-1, si-SNHG6-2 and si-SNHG6-3) were designed and assessed for their transfection efficiency using qRT-PCR. The efficiency of si-SNHG6-2 was higher than si-SNHG6-1 and si-SNHG6-3 in MDA-MB-231 and BT-549 cells, so it was utilized for subsequent experiments (**Figures 2A, B**). Inhibiting lncRNA SNHG6 diminished the proliferative abilities of two kinds of cells in contrast to cells transfected with the si-NC group (**Figures 2C, D**). Meanwhile, inhibiting lncRNA SNHG6 resulted in reduced formation ability of both cell lines (**Figures 2E, F**). These findings demonstrated that the suppressing of lncRNA SNHG6 expression slowed TNBC cell proliferation.

### lncRNA SNHG6 Inhibition Suppressed the Migratory Capability of TNBC Cells

We then evaluated the impact of lncRNA SNHG6 on the migratory abilities of TNBC cells. MDA-MB-231 and BT-549 cells were found to migrate at a slower rate on wound healing assay after lncRNA SNHG6 knockdown (**Figures 2G, H**). Similarly, lower numbers of migrating cells were noted upon lncRNA SNHG6 knockdown (**Figures 2I, J**).

### lncRNA SNHG6 Inhibition Promoted the Apoptosis Capability of TNBC Cells

Then we examined the effect of lncRNA SNHG6 on apoptosis ability of TNBC cells. The results showed that the number of TUNEL positive cells increased significantly after SNHG6 knock down in MDA-MB-231 and BT-549 cell lines (**Figures 2K, L**). Moreover, the protein expression of Bcl-2 decreased while the expression of Bax and c-Caspase-3 increased after SNHG6 knock down in two kinds of cell lines (**Figures 2M, N**).

### Knockdown of lncRNA SNHG6 Inhibited Tumorigenicity in Xenograft Model

**Figure 3:**
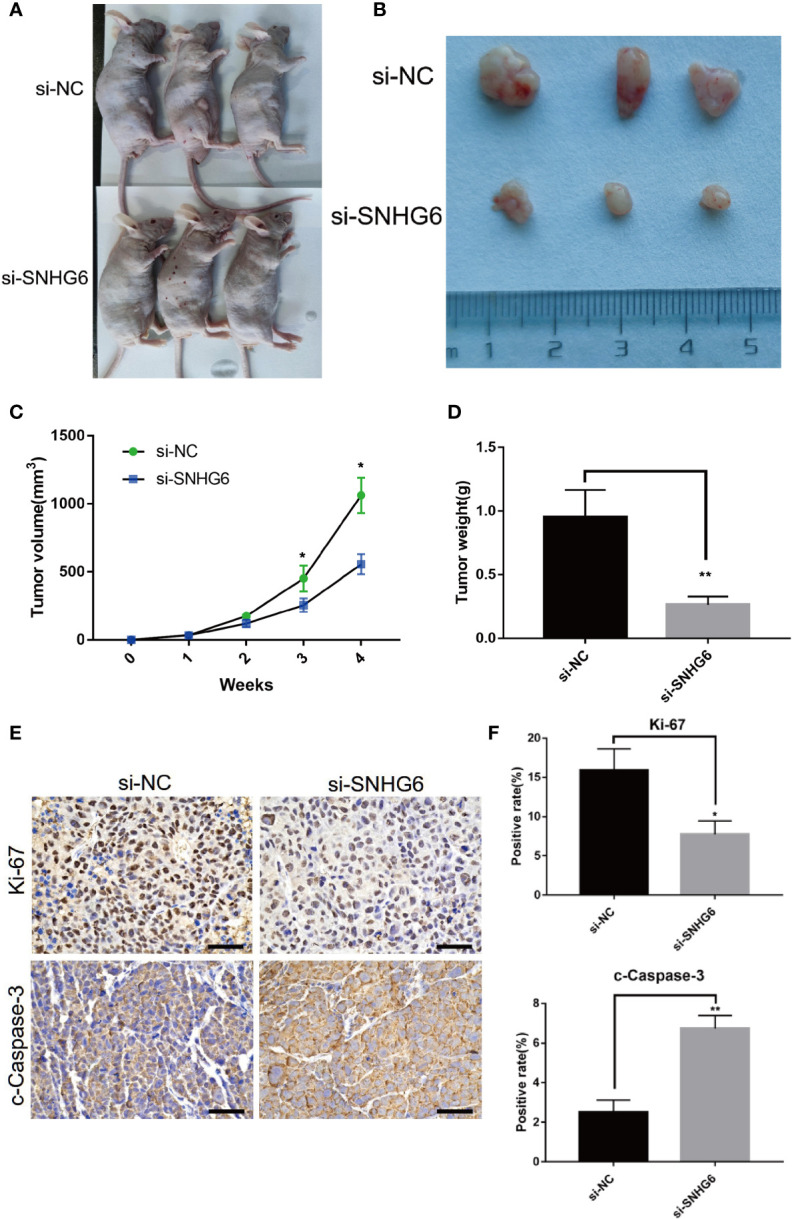
Knockdown of lncRNA SNHG6 inhibited tumorigenicity in xenograft model. **(A)** Representative images of a xenograft mouse model. **(B)** Representative tumor images. **(C)** Statistical results of tumor volumes. **(D)** Statistical results of tumor weights. **(E)** Representative Ki-67 and c-Caspase-3 staining images of tumor. **(F)** lncRNA SNHG6 inhibition suppressed the expression of Ki-67 and promoted the expression of c-Caspase-3 in xenograft model tissues. Magnification ×200; Scale bar, 100 μm.*P < 0.05, **P < 0.01 vs. miR-NC; n = 3.

To further detect the effect of lncRNA SNHG6 downregulation on TNBC growth *in vivo*, we established a xenograft mouse model with MDA-MB-231 cells. lncRNA SNHG6 expression was artificially downregulated in MDA-MB-231 cells with siRNA sequences targeting SNHG6. Knockdown of lncRNA SNHG6 markedly diminished tumor growth as shown by lower tumor weights and volumes (**Figures 3A–D**). Correspondingly, the expression of Ki-67 was lower in the si-SNHG6 group in contrast to that of the si-NC group while the expression of c-Caspase-3 was higher in si-SNHG6 group (**Figures 3E, F**). Our observations found that lncRNA SNHG6 knockdown inhibited the progression of TNBC in a xenograft mouse model.

### Downregulation of lncRNA SNHG6 Modulates Activity of the miR-125b-5p/BMPR1B axis in TNBC Cells

**Figure 4:**
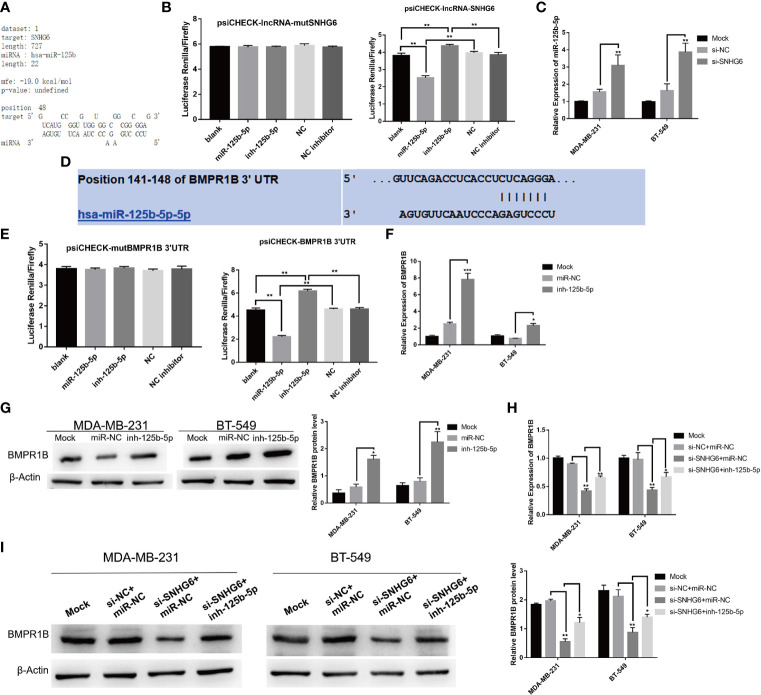
Downregulation of lncRNA SNHG6 regulates miR-125b-5p/BMPR1B signaling pathway in TNBC cells. **(A)** Predicted binding sites between lncRNA SNHG6 and miR-125b-5p. **(B)** Dual-luciferase reporter assay verified the presence of a direct binding site between miR-125b-5p and lncRNA SNHG6. **(C)** Knockdown of SNHG6 upregulated miR-125b-5p expression in MDA-MB-231 and BT-549 cells. **(D)** Predicted binding sites between miR-125b-5p and BMPR1B. **(E)** Dual-luciferase reporter assay verified the presence of a shared binding site between miR-125b-5p and BMPR1B. **(F)** miR-125b-5p inhibition upregulated BMPR1B mRNA expression. **(G)** miR-125b-5p inhibition upregulated BMPR1B protein expression. **(H)** miR-125b-5p inhibition attenuated the impact of lncRNA SNHG6 suppression on BMPR1B mRNA expression. **(I)** miR-125b-5p inhibition attenuated the impact of lncRNA SNHG6 suppression on BMPR1B protein levels. *P < 0.05, ** *P* < 0.01, *** *P* < 0.001; n = 3.

To fully characterize the molecular mechanism underlying lncRNA SNHG6, we used the RNAhybrid (https://bibiserv.cebitec.uni-bielefeld.de/rnahybrid/) program to predict potential lncRNA SNHG6 binding sites on miRNAs (19). miR-125b-5p was predicted to possess a binding site compatible with lncRNA SNHG6 (**Figure 4A**). The miR-125b-5p mimic was found to reduce luciferase activity of a reporter vector containing wild type SNHG6 but not those containing mutated SNHG6, as evidenced using a dual-luciferase reporter assay (**Figure 4B**). In addition, inhibiting lncRNA SNHG6 upregulated miR-125b-5p expression in MDA-MB-231 and BT-549 cells (**Figure 4C**).

Potential miR-125b-5p associated downstream molecules were predicted using the TargetScan Human 7.1 (http://www.targetscan.org/vert_72/) (20), miRDB (http://mirdb.org/) and miRanda (http://www.microrna.org/microrna/home.do) (21). The prediction results show that BMPR1B (which encodes the BMR1B protein) may be a potential target gene of miR-125b-5p (**Figure 4D**). Luciferase assay also found that BMPR1B was a direct target of miR-125b-5p (**Figure 4E**). Further miR-125b-5p inhibition upregulated BMPR1B at both mRNA and protein expression levels in MDA-MB-231 and BT-549 cells (**Figures 4F, G**).

We further validated the relationship between lncRNA SNHG6, miR-125b-5p and BMPR1B. The protein and mRNA expressions of BMPR1B were downregulated after lncRNA SNHG6 knockdown in MDA-MB-231 and BT-549 cells. Whereas, co-administration with miR-125b-5p inhibitor appeared to attenuate the impact of lncRNA SNHG6 suppression on BMPR1B expression in both cell lines (**Figures 4H, I**). This series of investigations strengthen our hypothesis that the lncRNA SNHG6/miR-125b-5p/BMPR1B axis plays a prominent role in TNBC cells.

### The Inhibition of miR-125b-5p Attenuated the Effect of lncRNA SNHG6 Knockdown on Proliferation, Migration, and Apoptosis of TNBC Cells

**Figure 5:**
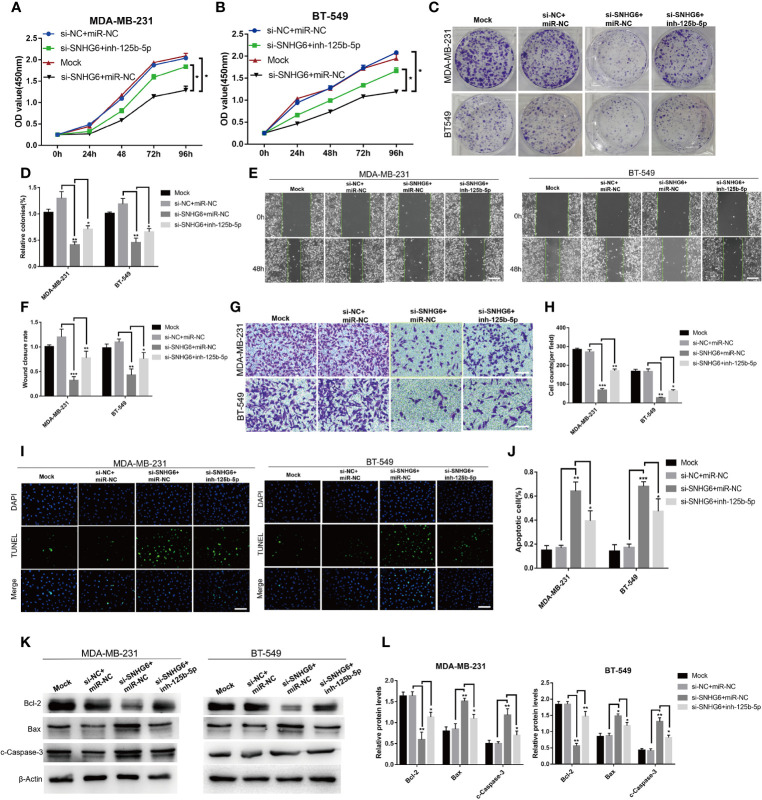
Inhibiting miR-125b-5p attenuates the impact of lncRNA SNHG6 suppression on TNBC cells. **(A)** miR-125b-5p inhibition attenuated the impact of lncRNA SNHG6 suppression on the proliferation of MDA-MB-231 cells. **(B)** miR-125b-5p inhibition attenuated the impact of lncRNA SNHG6 suppression on the proliferation of BT-549 cells. **(C)** Colony formation images of MDA-MB-231 and BT-549 cells. **(D)** miR-125b-5p inhibition attenuated the impact of lncRNA SNHG6 suppression on colony-formation capabilities of MDA-MB-231 and BT-549 cells. **(E)** Representative wound healing assay images of MDA-MB-231 and BT-549 cells. Magnification×40; Scale bar, 200 μm. **(F)** miR-125b-5p inhibition attenuated the impact of lncRNA SNHG6 suppression on the migratory capability of both two kinds of cells. **(G)** Boyden chamber cell migration assay in MDA-MB-231 and BT-549 cells, respectively. Magnification×100; Scale bar, 100 μm. **(H)** miR-125b-5p inhibition attenuated the impact of lncRNA SNHG6 suppression on the migratory capability of both two kinds of cells. **(I)** TUNEL assay in MDA-MB-231 and BT-549 cells, respectively. Magnification×100; Scale bar, 100 μm. **(J)** miR-125b-5p inhibition attenuated the impact of lncRNA SNHG6 suppression on the apoptosis capability of both two kinds of cells. **(K)** The protein expression of Bcl-2, Bax and c-Caspase-3 in MDA-MB-231 and BT-549 cells. **(L)** miR-125b-5p inhibition attenuated protein expression level of Bcl-2, Bax and c-Caspase-3 after SNHG6 knocked down. *P < 0.05, ** *P* < 0.01, *** *P* < 0.001 vs. si-NC; n = 3.

We then explored whether lncRNA SNHG6/miR-125b-5p/BMPR1B axis is involved in the growth of TNBC cells. The knockdown of lncRNA SNHG6 inhibited the ability of proliferation and migration, while promoted the ability of apoptosis in MDA-MB-231 and BT-549 cells. Whereas, the inhibition of miR-125b-5p attenuated the effect of lncRNA SNHG6 knockdown on proliferation, migration, apoptosis of above two kinds of cells (**Figure 5**). Therefore, lncRNA SNHG6/miR-125b-5p/BMPR1B pathway was involved in the growth of TNBC cells.

## Discussion

Breast cancer makes up approximately one-third of all cancers diagnosed in women (22). The current treatment for breast cancer, especially for TNBC, is still inadequate (23). LncRNAs may confer critical effects in breast cancer progression and development (24–26). Our study investigated the function of lncRNA SNHG6 in TNBC and explored its potential mechanism.

Previous studies found breast cancer tissues to harbor significantly higher expressions of lncRNA SNHG6 in contrast to adjacent normal breast tissues (27). Similarly, our study also found that breast cancer cell lines, particularly those of TNBC, had higher lncRNA SNHG6 expression profiles in contrast to normal breast epithelial cell line. We then observed the effect of lncRNA SNHG6 knockdown on MDA-MB-231 and BT-549 TNBC cells which were found to have higher endogenous expressions of lncRNA SNHG6. lncRNA SNHG6 knockdown suppressed the migratory and proliferative abilities, while promoted the apoptosis ability of TNBC cells. The vivo effects of lncRNA SNHG6 suppression on TNBC were also assessed. lncRNA SNHG6 downregulated resulted in lower tumor weights and volumes in a xenograft mouse model, as evidenced by lower Ki-67 and higher c-Caspase-3 expression in these tumor tissues. These findings indicate that downregulation of lncRNA SNHG6 could inhibit both *in vivo* and vitro growth of TNBC cells.

We further sought to predict the potential molecular mechanism of lncRNA SNHG6 using bioinformatic analyses. The prediction results showed that lncRNA SNHG6 and miR-125b-5p share similar binding sites. Both miR-125b-5p and lncRNA SNHG6 were found to directly interact, as performance by dual-luciferase reporter assays. There was elevated miR-125b-5p expression upon artificial lncRNA SNHG6 suppression. This finding is in line with previous studies which found that TNBC cells demonstrated marked downregulation of miR-125b-5p in contrast to normal breast tissues (28). Its expression was also lower in breast cancer cells (MCF-7, MDA-MB-231 and T47D) in comparison to non-tumorigenic epithelial cell line MCF-10A (29). miR-125b-5p was also postulated to function as a breast tumor suppressor (30). Breast cancer cell proliferative, migratory and invasive capabilities were suppressed upon miR-125b-5p overexpression (29). Furthermore, circulating miR-125-5p appeared to be helpful in breast cancer risk stratification (31). Therefore, the effect of lncRNA SNHG6 in breast cancer appears to be mediated by miR-125b-5p.

Further investigation found that BMPR1B may be a potential miR-125b-5p target. Raised BMPR1B expression may likely lead to enhanced MDA-MB-468 TNBC cell migration due to its effect in augmenting CYP2J2 expression (32). Genetic variations in BMPR1B binding sites have been linked to breast cancer risk (33). Moreover, the specific genetic variant involving the miR-125b—BMPR1B binding site has been found to contribute to breast cancer pathogenesis (34). We demonstrated that BMPR1B to be targeted by miR-125b-5p, with miR-125b-5p inhibition translating to raised BMPR1B expression in TNBC cells. Similarly, previous studies revealed that miR-125b promotes ovarian granulosa cell apoptosis through its action on BMPR1B (35).

Subsequently, the effect of lncRNA SNHG6 knockdown on BMPR1B expression was analyzed. Knocking down lncRNA SNHG6 resulted in decreased BMPR1B expression, however, the co-administration of a miR-125b-5p inhibitor partially reversed this finding. This highlights the fact that suppressing lncRNA SNHG6 inhibited BMPR1B expression and enhanced miR-125b-5p expression.

Finally, we detected whether lncRNA SNHG6/miR-125b-5p/BMPR1B axis was related to TNBC cell growth. The downregulation of lncRNA SNHG6 inhibited proliferation, migration and promoted apoptosis of MDA-MB-231 and BT-549 cells, which was attenuated through inhibiting miR-125b-5p.

To conclude, TNBC cells which possessed suppressed lncRNA SNHG6 levels also demonstrated attenuated migratory, proliferative, and promoted apoptosis ability and it is likely owing to the effect of miR-125b-5p/BMPR1B signal pathway modulation, with this axis representing a novel target in developing new treatments for TNBC.

## Data Availability Statement

The original contributions presented in the study are included in the article/supplementary material. Further inquiries can be directed to the corresponding authors.

## Ethics Statement

The animal study was reviewed and approved by The Harbin Medical University Animal Care and Use Committee.

## Author Contributions

YL and YZ conceived the idea of the study. HY and XQ analysed the data. XL, HY, XQ, XW, CL, XS, and HG interpreted the results. JZ and YL wrote the paper. All authors contributed to the article and approved the submitted version.

## Funding

This work was supported by the National Natural Science Foundation of China (81972469, 81803524 and 81803012) and Harbin Medical University Graduated Research and Practical Innovation Program (YJSKYCX2019-04HYD).

## Conflict of Interest

The authors declare that the research was conducted in the absence of any commercial or financial relationships that could be construed as a potential conflict of interest.
